# Human Umbilical Cord Matrix Stem Cells Maintain Multilineage Differentiation Abilities and Do Not Transform during Long-Term Culture

**DOI:** 10.1371/journal.pone.0071374

**Published:** 2013-08-09

**Authors:** Isabelle Scheers, Catherine Lombard, Massimiliano Paganelli, David Campard, Mustapha Najimi, Jean-Luc Gala, Anabelle Decottignies, Etienne Sokal

**Affiliations:** 1 Université Catholique de Louvain, Institut de Recherche Expérimentale et Clinique (IREC), Laboratory of Pediatric Hepatology and Cell Therapy, Brussels, Belgium; 2 Université Catholique de Louvain, Institut de Recherche Expérimentale et Clinique (IREC), Center for Applied Molecular Technologies, Brussels, Belgium; 3 Université Catholique de Louvain, de Duve Institute, Genetic and Epigenetic Alterations of Genomes Unit, Brussels, Belgium; Scuola Superiore Sant'Anna, Italy

## Abstract

Umbilical cord matrix stem cells (UCMSC) have generated great interest in various therapeutic approaches, including liver regeneration. This article aims to analyze the specific characteristics and the potential occurrence of premalignant alterations of UCMSC during long-term expansion, which are important issues for clinical applications. UCMSC were isolated from the umbilical cord of 14 full-term newborns and expanded *in vitro* until senescence. We examined the long-term growth potential, senescence characteristics, immunophenotype and multilineage differentiation capacity of these cells. In addition, their genetic stability was assessed through karyotyping, telomerase maintenance mechanisms and analysis of expression and functionality of cell cycle regulation genes. The tumorigenic potential was also studied in immunocompromised mice. *In vitro*, UCMSC reached up to 33.7±2.1 cumulative population doublings before entering replicative senescence. Their immunophenotype and differentiation potential, notably into hepatocyte-like cells, remained stable over time. Cytogenetic analyses did not reveal any chromosomal abnormality and the expression of oncogenes was not induced. Telomere maintenance mechanisms were not activated. Just as UCMSC lacked transformed features *in vitro*, they could not give rise to tumors *in vivo*. UCMSC could be expanded in long-term cultures while maintaining stable genetic features and endodermal differentiation potential. UCMSC therefore represent safe candidates for liver regenerative medicine.

## Introduction

Stem cell transplantation has recently emerged as a promising alternative approach to orthotopic liver transplantation for the treatment of metabolic liver diseases [1], [2]. Among the potential candidates, umbilical cord matrix stem cells (UCMSC) have received a great deal of interest owing to their multipotency, proliferation potential and low immunologic profile. In addition, UCMSC stand as a non-controversial and easily accessible cell source for clinical-based therapies. The multipotency of UCMSC has been widely demonstrated *in vitro* and *in vivo* in animal models. Among others, they have shown the ability to differentiate into osteoblasts [3], adipocytes [4] and even neurons [5]. We have further reported their ability to acquire specific hepatocyte-like functions [6] making them suitable for liver-based cell therapies [7]. Furthermore, UCMSC present interesting tolerogenic properties for allogenic cell transplantation [8].

Encouraging results regarding the efficacy of a large range of cell-based therapies in animal models rapidly stimulated investigators to move towards clinical trials, thus requiring broader investigations on safety concerns. The clinical development of UCMSC requires cell isolation and *in vitro* expansion. *Ex vivo* culture can potentially alter cell properties, induce DNA damage and checkpoint activation; leading to premature senescence [9], [10]. In contrast with various stem cells isolated from animal models [11], [12], transformation of human mesenchymal stem cells is a rare – although described – event [13]. Nonetheless, carcinogenicity of long-term cultured human stem cells is a matter of controversy. Indeed, few authors observed spontaneous transformation in human derived MSC. However, most of those studies were later retracted as the presented results could not be reproduced or were explained by culture cross contamination [14], [15]. In contrast, other groups, studying the same cells, could not evidence any sign of cell transformation. Mechanisms driving human cell transformation *in vitro* seem to involve cytogenetic instability [16], oncogene activation, defective checkpoint control and telomere stabilization [17], [18]. Noteworthy, although this has not been established yet, the immunotolerogenic features of these cells [8], [19] could impair patient’s antitumoral responses towards transformed cells. As the available information on the risk of human cell transformation remains scarce, despite the concern of clinicians, it is of primary importance to carefully characterize UCMSC after *in vitro* expansion.

In the present study, we isolated and cultured MSC from Wharton’s jelly for large-scale preclinical batch testing. *In vitro* long lasting phenotype stability and differentiation potential were investigated in cells cultured until senescence. Furthermore, we evaluated the chromosomal stability, functionality of genes involved in cell cycle regulation, and activation of telomere maintenance mechanisms. Finally, the *in vivo* tumorigenic potential of UCMSC was assessed by subcutaneous injection in an immunocompromised xenograft model.

## Methods

### UCMSC Isolation, Cell Lines and Culture

The present study was approved by the institution’s ethical committee (Cliniques Universitaires Saint-Luc, Brussels, Belgium) and performed for preclinical testing purposes. Umbilical cords were collected, after written informed consent, from patients delivering full term infants (n = 14) and matrix stem cells were isolated as previously described [6]. Cells were seeded at a density of 7500 cells/cm^2^ and cultured in Dulbecco's modified Eagle medium (DMEM 1 g/l; Invitrogen, Merelbeke, Belgium) supplemented with 10% fetal bovine serum (FBS; PAA Laboratories GmbH, Pasching, Austria) and antibiotics (100 U/ml penicillin, 100 µg/ml streptomycin; Invitrogen). The medium was replaced twice per week and cells were detached when reaching 70% confluence. Population doubling (PD) was calculated using the following equation: [log_10_(N_H_)-log_10_ (N_I_)]/log_10_(2), where N_I_ is the inoculum number and N_H_ is the harvested cell number. The cumulative population doubling (CPD) was calculated by adding the PD obtained at each successive passage. The population doubling time (PDT) was calculated using the equation: PD/T, where T (in hours) is the time between cell seeding and harvesting. Cells were measured in suspension using the Axioscop microscope and software (Zeiss, Zaventem, Belgium). U2OS (ATCC, Manassas, USA) and HeLa cancer cell lines were kindly provided by Pr C. Sybille (Center for Human Genetics, Brussels, Belgium). HepG2 hepatoblastoma cell line was purchased from ATCC. Cancer cell lines were cultured in DMEM high glucose (4.5 g/l; Invitrogen) supplemented with 10% FBS and antibiotics. For HepG2 cells, 1% sodium pyruvate and 1% non-essential amino acids (both from Invitrogen) were added to the medium. Human hepatocytes were isolated, after written informed consent of the next of kin, from liver graft segments of 2 newborn donors (4 and 6 days of age, respectively) according to a protocol described elsewhere [20].

### Cell Surface Marker Analyses by Flow Cytometry

Every three passages, cells were harvested and incubated for 30 minutes with antibodies against hematopoietic markers (CD14-FITC, CD34-PE, CD117-APC, CD45-PE-Cy), mesenchymal stem cell markers (CD73-PE, CD90-APC, CD105-FITC), integrins and receptors for extracellular matrix components (CD29-APC, CD44-FITC, CD49e-PE, CD146-PE) and Cytokeratin-FITC (all from BD Bioscience, New Jersey, USA; except CD105 provided by Ancell, Bayport, USA) following previously described technique [6]. The corresponding isotypes (BD Bioscience) were used for evaluation of nonspecific binding. Cells were analyzed on a FACS Canto II flow cytometer using the FACSDiva software (BD Bioscience), and a minimum of 10,000 events were acquired for each sample.

### Immunofluorescence

Cells cultured on collagen type I-coated round glass coverslips were analyzed by immunofluorescence staining using standard protocols. Cells were incubated overnight with the following primary antibodies: vimentin (1/2000; Sigma, Bornem, Belgium), α-smooth muscle actin (ASMA, 1/2500; Chemicon, Hants, UK), cytokeratin 19 (1/2000; Chemicon). A Cy3-coupled goat anti-mouse antibody (1/2000; Jackson, Newmarket, UK) was used as secondary antibody. Nuclei were stained with 4', 6-diamidino-2-phenylindole (1/5000; Sigma). Preparations were mounted with FluoPrep (BioMerieux, Brussels, Belgium) and examined with a fluorescence microscope (Axioscop, Zeiss). To study telomere dysfunction induced senescence, we analyzed TRF2 and 53BP1 co-localization [21]. Cells were cultured on culture slides (BD Bioscience). Fixed cells were incubated with a mouse anti-human TRF2 primary antibody (1/500; Imgenex, San Diego, USA) and with a rabbit anti-human 53BP1 antibody (1/250; Novus Biologicals, Cambridge, UK) overnight at 4°C. Subsequently, the cells were incubated with an Alexa-488 goat anti-mouse and an Alexa-685 donkey anti-rabbit secondary antibody (both 1/1000; Invitrogen). Cells were mounted using the ProLong Gold with DAPI reagent (Invitrogen). Images were acquired using an Axiovert microscope, Apotome and Axiovision 4.6.3 software (Zeiss).

### Multilineage Differentiation Potential

The osteogenic, adipogenic and hepatogenic differentiation potentials were studied every three passages, following culture in appropriate differentiation medium, as previously described [6]. Calcium deposition was evaluated by Alizarin Red staining and lipid vesicles were revealed by Oil Red O staining. The osteocyte differentiation ability was expressed by quantifying the mineralized area using ImageJ software. The extent of adipogenic differentiation was assessed by counting cells containing oil droplets in 3 different random fields of 3 different culture samples at selected passages [22]. Hepatic functional testing included measurement of: a) glycogen storage using periodic acid Schiff’s staining (Sigma) followed by alpha amylase treatment, b) urea production with a colorimetric assay (Gentaur, Brussels, Belgium) and c) cytochrome P450 3A4 (CYP3A4) metabolic activity using a luciferase assay (Promega, Leiden, The Netherlands).

### Senescence Associated-β-galactosidase Staining

UCMSC from each passage were seeded at a density of 1.10^4^/cm^2^ in 6-well plates. After 24 h, cells were fixed in 3.5% formaldehyde, and subjected to Senescence associated-β-galactosidase (SA-β-Gal) staining [23] following the manufacturer’s protocol (Sigma).

### Soft Agar Assay

Cells from passages (P)9 and 18 were plated in triplicate in DMEM medium supplemented with 20% FBS and 0.3% Agar Noble (Sigma) as previously described [24]. Additional medium (1 ml) was added twice a week. After 6 weeks, colonies of more than 10 cells were counted under an optical microscope. HepG2 cells were used as a positive control.

### Cell Proliferation in Serum Free Conditions

Cells from every three passages were seeded in triplicate at a density of 8.10^3^/cm^2^ in 96-well plates. After 24 h, the medium was replaced with serum free medium for 7 days. Cell proliferation was evaluated by adding 1 µCi/well (^3^H)thymidine (MP Biomedicals, Irvine, USA) during the last 24 h. Radioactive uptake was measured using a TopCount NXT microplate scintillation counter (Packard Bioscience, Meriden, USA). HepG2 cells and 40 gray γ-irradiated cells were used as positive and negative control respectively.

### Telomerase Activity and Telomere Length Assay

Detection of telomerase activity was performed using the Trapeze Telomerase RT Detection kit (Chemicon). Telomere length was analyzed with telo TAGGG Telomere Length assay kit (Roche, Mannheim, Germany) as described previously [25].

### p53 Functionality Test and Detection of Cell Cycle-related Proteins

Functional analysis of separated alleles in yeast (FASAY) was performed to evaluate p53 activity [26]. At least 100 colonies were counted per sample. Red yeast colonies indicate a mutated *p53* allele, however the presence of less than 10% red colonies was considered as wild-type because of PCR-induced errors or the presence of an alternatively spliced *p53* mRNA [27]. A blood sample from a Li-Fraumeni patient (mutated p53) was used as a positive control.

p21, p16, p53, pRb and c-Myc protein levels were analyzed on cell lysates by western blotting using standard procedures. Blots were probed with the following antibodies: anti-p21 (1/2000; Calbiochem, Darmstadt, Germany), anti-p16 (1/1000; Santa Cruz, Heidelberg, USA), anti-c-Myc (1/2500; Cell Signaling Technologies, Danvers, USA), anti-pRb and anti-p53 (1/2000; BD bioscience). All blots were also probed with mouse anti-α-actin (1/25000; Sigma). Immunoreactive proteins were quantified by densitometry using the GelDoc 2000 device and software (BioRad).

### Gene Expression Analyses

Total RNA was extracted from 1.10^6^ cells using TriPure isolation reagent (Roche). Following DNAse treatment (Invitrogen), cDNA was generated from 1 µg RNA using the Thermoscript RT kit (Invitrogen). PCR amplifications were performed in an iCycler (Applied Bioscience) using qPCR Mastermix (Applied Bioscience) and the following Taqman Probes (Applied Bioscience): TDO: Hs 00194611_m1; Cyp3A4: Hs 00604506_m1; Albumin: Hs 0060944_m1; GAPDH: Hs 99999905_m1; hTERT [28].

### Molecular Karyotyping and Fluorescence In Situ Hybridization (FISH)

Cytogenetic analyses were performed at P9 and P18. Chromosomes were G-banded and subsequently examined under optical microscopy. 200 metaphases were counted independently by two investigators, following the International System for Human Cytogenetic Nomenclature (ISCN) 2009 [29]. FISH was performed on either interphase or metaphase nuclei from P9–P18 UCMSC using probes for TP53 (Vysis TP53, Abbott, Diegem, Belgium), *EWSR1* (Vysis break apart probe LSI EWSR1), *BCR* (Vysis LSI 22) and 22q13 subtelomere (TelVysion22q).

### *In vivo* Tumorigenicity Assay

All animal experiments were performed in compliance with Belgian laws for animal protection, and approved by the local ethical review board (Université Catholique de Louvain, Brussels, Belgium - Permit Number: LA2230397). 10^7^ cells harvested at P9 or HepG2 tumor cells were subcutaneously injected into 4-week old Balb-c NuNu mice (Charles River Laboratories, Brussels, Belgium). The mice were monitored daily and were euthanized when the tumor volume reached 1 cm^3^, or after 24 weeks. Skin and organs were fixed in 3.5% formaldehyde for further analyses.

### Statistical Analyses

Statistical analyses were performed using Prism 4.1 (GraphPad software, San Diego, USA). To address the question of multiple comparisons, one-way Anova followed by Bonferroni correction was used. (***p<0.001, **p<0.01, *p<0.05). Results were presented as means±SEM.

## Results

### UCMSC Isolation and Characterization

**Figure 1:**
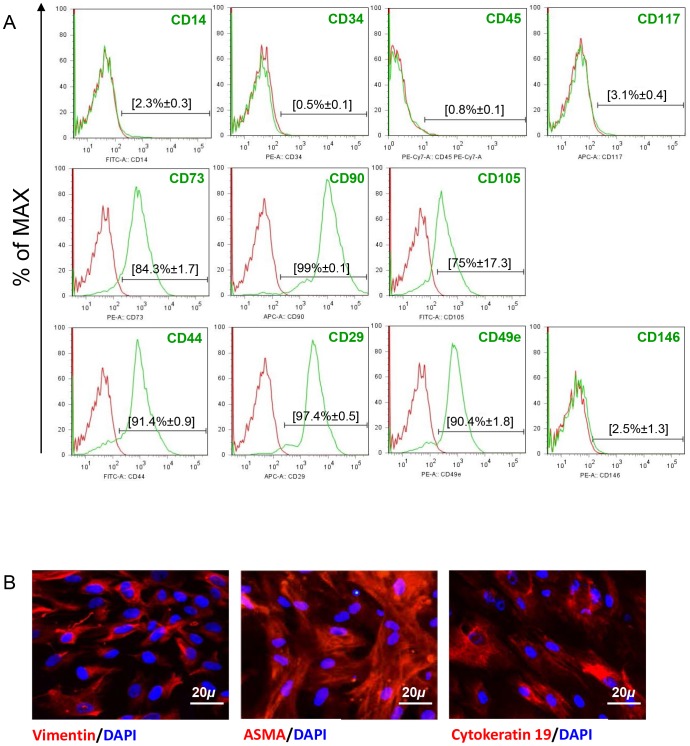
UCMSC display mesenchymal features. UCMSC derived from 14 different donors were characterized. A. Representative example of fluorescence-activated cell sorting assay performed on cells at P9. Surface marker expression is shown as a histogram (green) and compared to corresponding control isotype (red). Results are expressed as the mean±SEM percentage of positive cells for 14 independent experiments. B. Immunofluorescence staining for vimentin, ASMA and cytokeratin 19 in UCMSC at P9.

UCMSC samples were isolated from Wharton’s jelly of 14 different donors and checked for the expression of mesenchymal markers. A large portion of UCMSC expressed CD73 (84.3%±1.7), CD90 (99%±0.1) and CD105 (75%±17.3) (Figure 1A). The cells were also analyzed for extracellular matrix or cell-to-cell interaction markers such as CD29 (97.4%±0.5) and CD44 (91.4%±0.9). Contamination of the culture with hematopoietic cells was negligible: CD14 (2.3%±0.3), CD34 (0.5%±0.1), CD45 (0.8%±0.1) and CD117 (3.1%±0.4) expression. Similarly, the endothelial and progenitor marker CD146 was not expressed (2.5%±1.3). The mesodermal lineage of UCMSC was further confirmed by detection of ASMA and vimentin transcripts (data not shown) and proteins (Figure 1B). This phenotype remained stable throughout cell culture.

### Growth Potential and Entry into Senescence of UCMSC are Homogeneous between Donors

**Figure 2:**
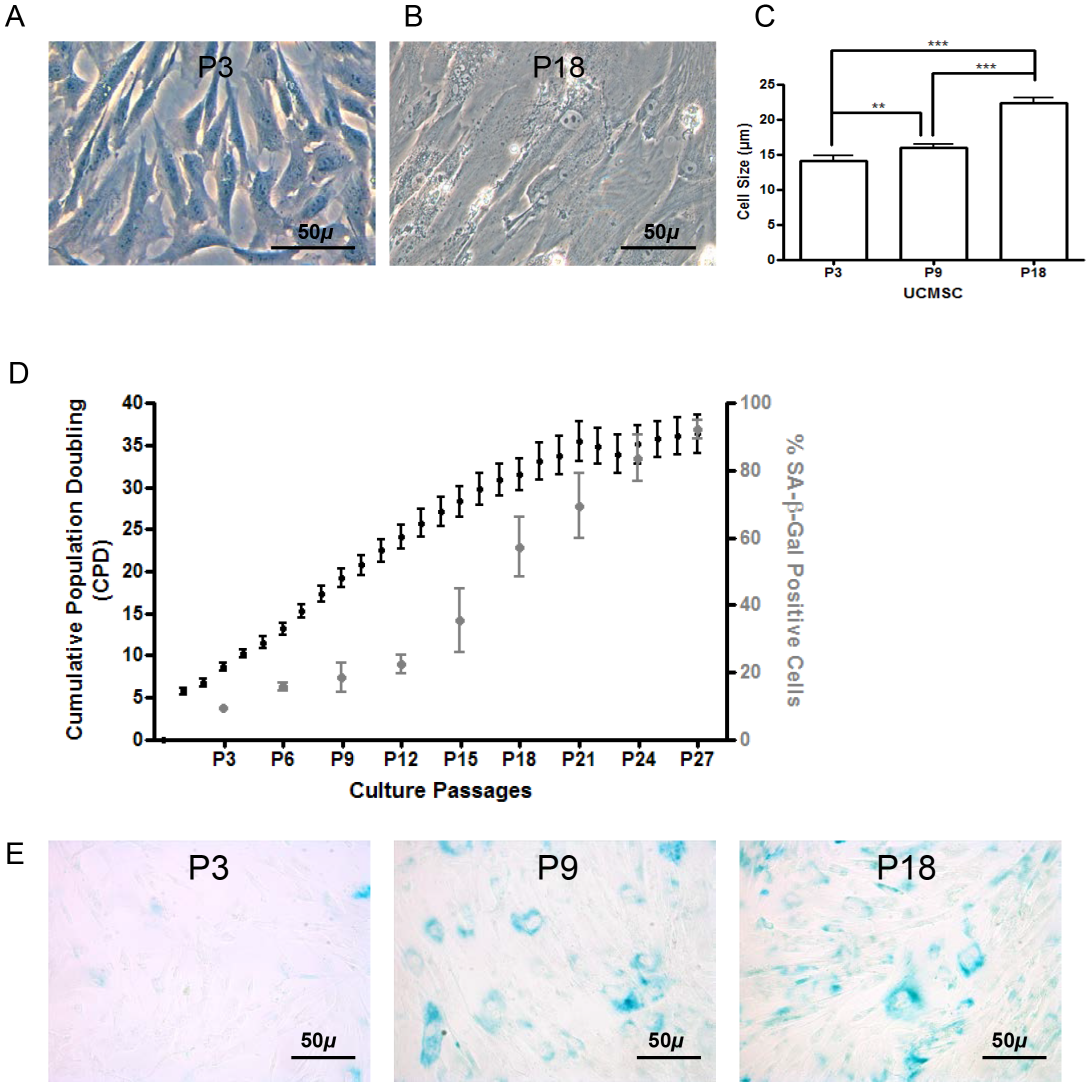
Growth potential and senescence features during long-term culture of UCMSC. A. At early passages (P3), UCMSC displayed a fibroblast-like morphology. B. With cell aging (P18), UCMSC became larger in size, with a more spindle-shape and flattened appearance. C. Average cell size of UCMSC in suspension measured using a confocal microscope at P3, P9 and P18. D. Growth kinetics of long-term cultured UCMSC. Cumulative population doubling (Y-axis left) is plotted against culture passage. Percentage of UCMSC staining positive for SA-β-gal (Y-axis right) at successive passages. E. SA-β-gal staining remained almost stable during early culture passages but increased significantly between P15 and P21.

UCMSC from the 14 donors were cultured *in vitro* until cell senescence. At early passages (P), cells had a small (average diameter of UCMSC in suspension at P3 of 14.1±0.7 µm) spindle shape appearance (Figure 2A). With ageing, cells progressively became more flattened and their size increased (22.3±0.8 µm at P18 - Figure 2B–C). UCMSC growth potential was homogeneous between donors with a phase of fast growth during the first fifteen passages and a mean population doubling time (PDT) of 3.1±0.4 days (Figure 2D). Cellular senescence, characterized by a decline in proliferation and final growth arrest [30], was reached at a mean cumulative population doubling (CPD) of 33.7±2.1 corresponding to 160.9±6.9 days of culture and 21.7±1.0 passages. Accordingly, the number of cells that stained positive for SA-β-Gal stayed around 5 to 10% during early culture passages and then increased markedly between P15 (38.2%±9.4) and P21 (71.5%±8.8) (Figure 2D–E). Although 13 out of the 14 UCMSC cultures stopped growing after they reached senescence, the culture issued from donor 4 transiently produced a cell cluster 5 weeks after entering senescence. Cells displayed cuboïdal morphology with a high nucleus to cytoplasm ratio. Flow cytometry results were typical for UCMSC and cells remained anchorage dependent in soft agar (Figure S1A–C). These cells could not be further propagated as they progressively detached from the flask after two weeks.

### UCMSC Retain *in vitro* Mesodermal and Endodermal Differentiation Potential after Long-term Culture

**Figure 3:**
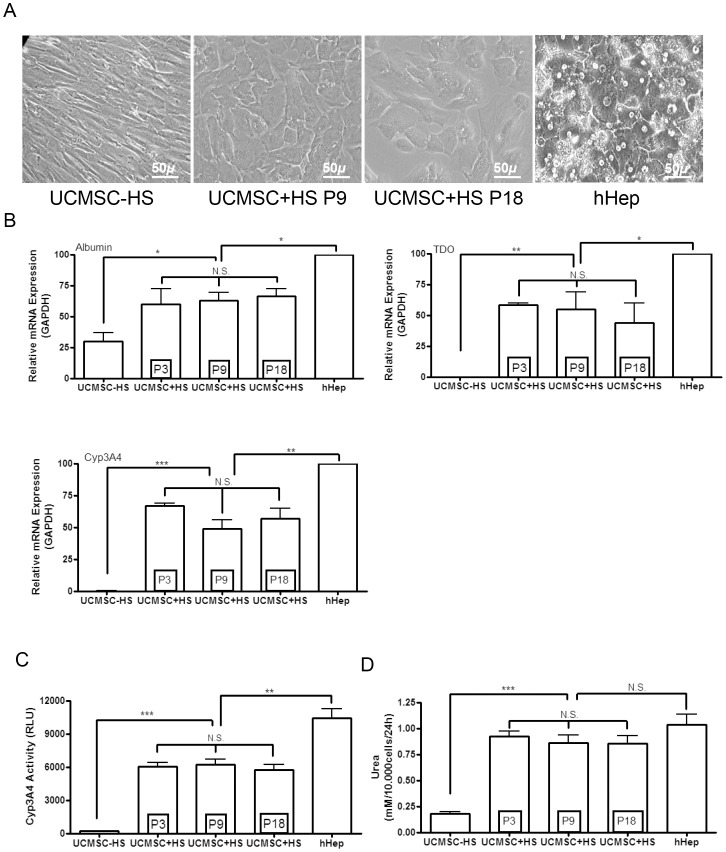
Multilineage *in vitro* differentiation potential of UCMSC. A. Optical microscopy pictures showing representative (n = 14) undifferentiated (−HS) and hepatocyte-like differentiated (+HS) UCMSC at P9 and P18. Cells acquired a rounded, polygonal shape with a granular cytoplasm and a central nucleus. Normal adult human hepatocytes (hHep) are shown for comparison. B. Graphs of qPCR analyses showing expression of mature hepatic marker genes in hepatocytes (hHep) and undifferentiated (−HS) or differentiated (+HS at P3, P9 and P18) UCMSC. Results were normalized to GAPDH. Differentiated UCMSC acquired hepatocyte-like functions, as confirmed by the induction of CYP3A4 activity (C) and urea production (D). Results of CYP3A4 activity are expressed as luciferase activity in relative luminescence unit (RLU). The urea production ability was expressed in milliMole (mM) of urea produced by 10.000cells over 24 hours.

UCMSC are known to display multilineage differentiation ability [3]. Previously, bone marrow MSC were reported to show a decreased differentiation ability with aging [31], [32], which could hamper tissue regeneration by transplanted cells. Here, we evaluated the *in vitro* differentiation efficiency of UCMSC from early and late passages into hepatocyte, osteocyte and adipocyte lineages, in order to assess whether senescence may affect their differentiation potential. When cultured in a differentiation medium that mimics the hepatic developmental milieu, UCMSC at either P3, P9 or P18 displayed progressive morphological changes into hepatocyte-like cells. Cells lost their spindle aspect to acquire a polygonal shape and displayed increased cytoplasmic granulations (Figure 3A). These morphological features correlated with the acquisition of markers and metabolic functions characteristic of hepatocytes. Indeed, expression of the albumin, tryptophan-2,3-dioxygenase (TDO) and CYP3A4 genes were similarly upregulated after differentiation of UCMSC at P3, P9 and P18 and reached levels close to those measured in hepatocytes (Figure 3B). Also, CYP3A4 activity was similarly acquired during the *in vitro* differentiation process (Figure 3C). Urea production was also significantly induced, nearly reaching the levels produced by hepatocytes (Figure 3D). Periodic acid-Schiff staining further showed that differentiated UCMSC were able to store glycogen (Figure S2A). However, UCMSC hepatocyte-like differentiation was no longer obtained after P18.

In agreement with the results obtained for hepatocyte differentiation, long-term cultured UCMSC retained osteogenic differentiation abilities up to P18, but was not further (Figure S2B). Similarly, the adipogenic differentiation potential was demonstrated at P3, P9 and P18. Afterwards, the number of small lipid droplets formed in the cytoplasm significantly decreased (Figure S2C).

### Chromosomal Stability is Maintained during Long-term Culture of UCMSC

**Figure 4:**
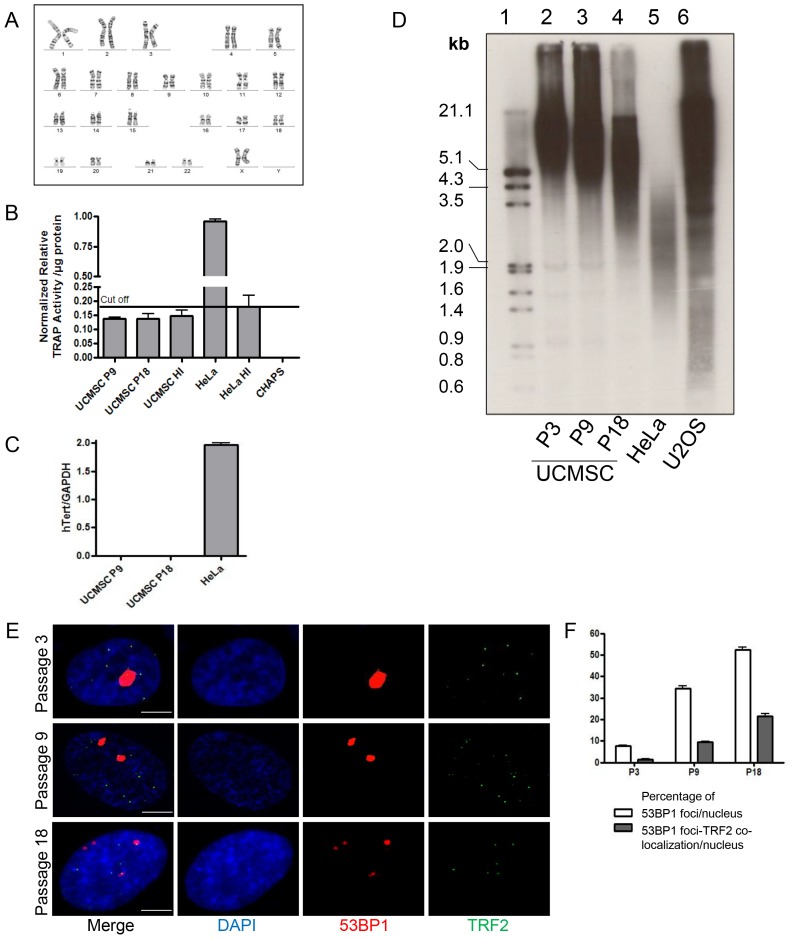
Karyotype and analysis of telomere maintenance mechanism. A. Representative normal G-banded karyotype of cultured UCMSC. B. Relative TRAP activity per microgram of protein lysate. Telomerase activity of UCMSC remained below the positive cut-off represented by heat inactivated (HI: 85°C for 10 min) HeLa cell extracts. C. Quantitative PCR confirmed the absence of *hTERT* gene transcripts in UCMSC at P9 and P18. HeLa cell cDNA was used as a positive control. D. Representative Southern blot analysis of telomere length. Lane 1 represents molecular weight markers. Telomere length of UCMSC shortened gradually between P3 and P18 (lane 2–4). Control HeLa cells (lane 5) displayed short telomeres, typical of most telomerase-positive cancer cell lines, while U2OS cells (lane 6) displayed the typical long and heterogeneous TRF pattern of ALT-positive cells. E. Cultured UCMSC display signs of telomere dysfunction induced cell senescence. Cells from P3, P9 and P18 were immunostained for 53BP1 DNA damage marker (red) and TRF2 telomeric protein (green). Co-localization of 53BP1 foci with TRF2 was scored on at least 50 nuclei to determine telomere dysfunction induced DNA damage foci (TIF). F. Quantification (%) of nuclei presenting 53BP1 foci at P3, P9 and P18. Percentage of nuclei presenting co-localization of 53BP1 with TRF2 in UCMSC at selected passages. (Scale bar 5 µm).

Long-term culture is a non-physiological process. Outside their natural niche, cells are exposed to different culture-related stresses and undergo multiple divisions that may potentially trigger chromosomal instability [8]. To check whether long-term culture of UCMSC impacted on their genomic stability, we performed karyotype analyses at P9 and at senescence. Karyotypes were found to be normal for 13/14 culture samples at both time points (Figure 4A). In the remaining sample, we identified a polymorphism of chromosome 22 (Figure S3A–B). Fluorescent in situ hybridization (FISH) was performed to analyze loss of heterozygosity in oncogenic relevant regions of chromosome 22. We could not detect bcr-abl translocation or abnormalities of *ESWR1* locus (Figure S3B-C). These observations suggested a donor-related particularity without any oncogenic connotation.

### UCMSC do not Activate Telomere Maintenance Mechanisms in Culture

UCMSC were tested for the activation of telomerase at P3, P9 and at senescence using TRAP assay and detection of telomerase reverse transcriptase (h*TERT*) transcripts by qRT-PCR. We failed to detect telomerase activity (Figure 4B) and h*TERT* gene transcription (Figure 4C) at all time points and in all samples examined. To further rule out the possibility that an alternative mechanism of telomere lengthening (ALT) was activated, we analyzed telomeres by Southern blotting (telomere restriction fragment, TRF) using a telomeric probe. The telomeres of UCMSC at P3 displayed a homogenous length that decreased with cell passaging (Figure 4D). Importantly, we failed to detect the appearance of a heterogeneous telomere length profile characteristic of ALT cells as shown for the control U2OS sarcoma cell line. Supporting telomere erosion with successive passages of UCMSC, we found an increased frequency of telomere dysfunction-induced foci (TIF), detected by co-localization between 53BP1 DNA damage marker and TRF2 telomeric protein (Figure 4E–F). Our immunofluorescence analyses also revealed an increased frequency of DNA damage foci outside telomeres at later passages, suggesting that telomeres may not be the only genomic loci undergoing damage during cell culture.

### Expression of Cell Cycle Regulation Genes and Oncogenes during Long-term Culture

**Figure 5:**
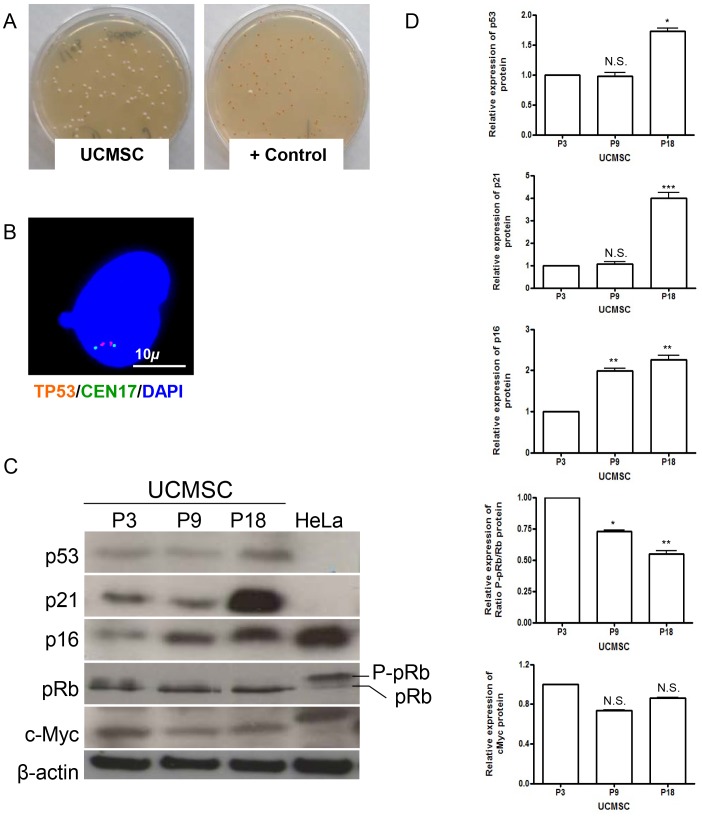
FASAY, cell cycle regulation proteins and oncogene activation in UCMSC. A. FASAY results analyzing the *TP53* gene isolated either from UCMSC or *p53*-mutated cells issued from a Li-Fraumeni patient. White yeast colonies are indicative of a wild-type *p53* allele while mutated *p53* induces red colony formation. B. Representative interphase FISH analysis of the TP53 locus confirming the presence of both gene alleles in the 17p13.1 region. C. Western blot analysis of p21, p53, p16, pRb and c-Myc proteins in UCMSC at different passages (P3, P9 and P18). Cervix cancer HeLa cells were used as a control. p21 was not detected in HeLa cells, while both c-Myc and phosphorylated pRb were up-regulated. The high level of p16 protein in HeLa cells was previously reported [56] but does not impair cell growth because pRb is inhibited by E7 papillomavirus protein in that cell line. The c-Myc protein of HeLa cells is of higher molecular weight because of the insertion of a mitochondrial DNA fragment in the *c-Myc* locus [57].

We next investigated the expression and functionality of p16/pRb and p53/p21, two pathways involved in cell cycle regulation, balancing tumor suppression and stem cell aging [33], [34], [35]. The majority of TP53 mutations are missense mutations leading to a significant loss of DNA binding and transactivation [36]. The impact of the mutation is determined by location and type of amino acid alteration. In addition, some missense mutations have been reported to display gain-of-function properties causing an oncogenic effect in carcinogenesis [37], [38], [39]. Nonsense mutations result in a truncated, usually nonfunctional protein. Also, insertions and deletions of one or more nucleotides that cause a frameshift and mutations at splice sites usually lead to a truncated protein [40]. To specifically analyze these situations, the expression and function of p53 were assessed by FASAY, FISH and Western blotting. The heterozygosity of *TP53* gene was confirmed by FISH, as cells presented two alleles of the gene on chromosome 17 (n = 100 mitosis) (Figure 5A). The functionality of p53 was normal in all tested populations, with a FASAY showing less than 10% red colonies (Figure 5B). Western blot analysis revealed that the levels of p53 tumor suppressor were up-regulated in UCMSC P18 samples and, consequently, the levels of p21 protein increased markedly (Figure 5C). The levels of p16 also rose progressively with long-term culture. Consequently, the phosphorylated state of pRb, was present in early cultures but vanished at senescence. Furthermore, UCMSC displayed low levels of the proto-oncogene c-Myc that even tended to decrease with cell aging.

### UCMSC are not Tumorigenic in both *in vitro* and *in vivo* Assays

**Figure 6:**
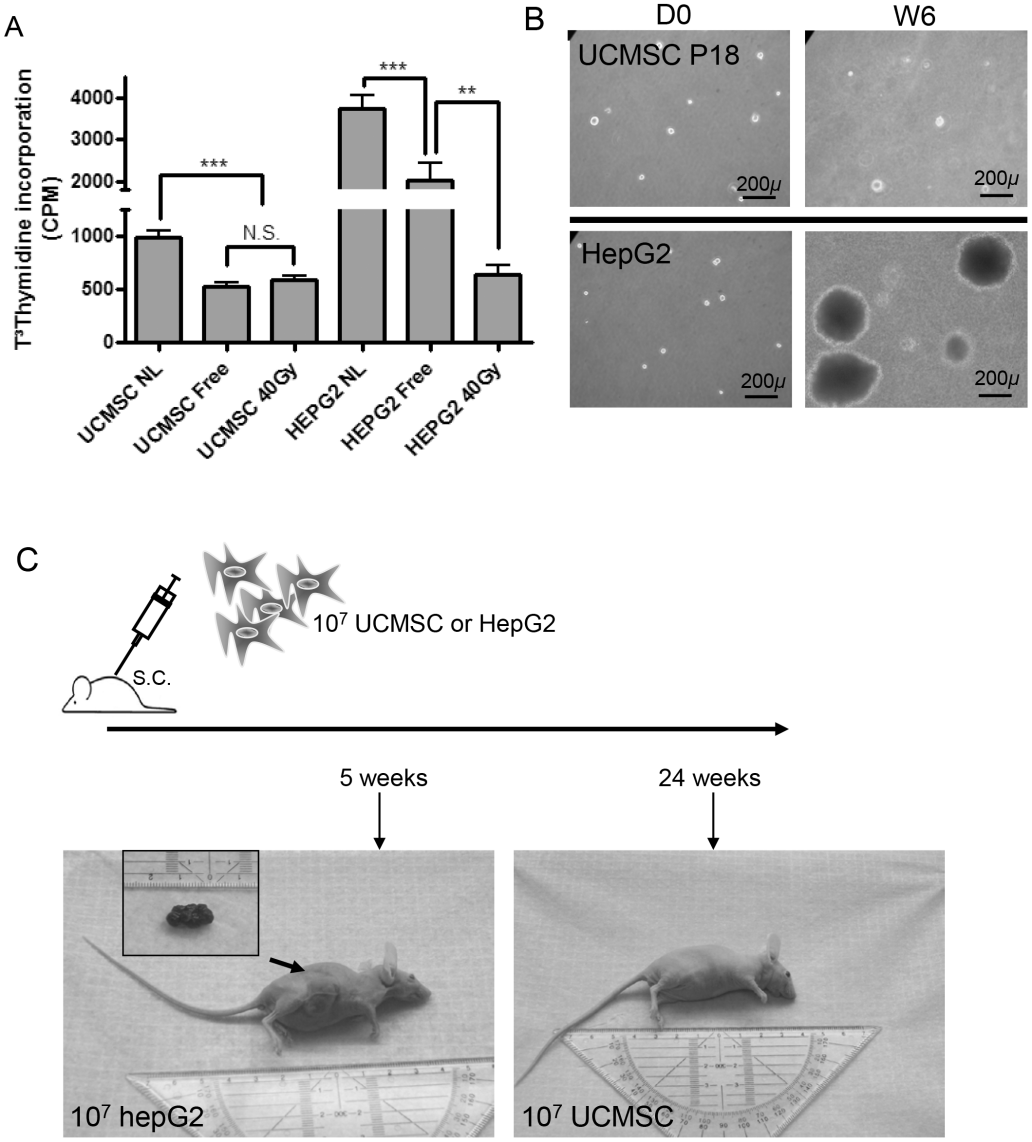
UCMSC do not display tumorigenic properties *in vitro* or *in vivo.* A. UCMSC could not proliferate in serum-deprived medium in contrast to HepG2 cells that displayed acquired self-sufficiency in growth signals. Cells grown in complete medium (NL) and in serum free medium (Free) were compared with negative controls obtained after γ-irradiation with 40Gy. B. UCMSC (P9 and P18) seeded in 0.3% Agar Noble were not able to form colonies. Representative experiment for n = 14 UCMSC samples tested at P18, after 1 day (D1) and 6 weeks (W6) in culture. HepG2 served as controls for each experiment and demonstrated clonal expansion. C. Balb-c/Nu mice were injected subcutaneously with 10^7^ HepG2 or UCMSC cells. Tumor growth was observed macroscopically in mice inoculated 5 weeks earlier with HepG2 cells (left) while UCMSC were not tumorigenic (right).

The analysis of both oncogene and cell cycle regulators that we carried out in UCMSC did not reveal any clue that cells were acquiring a tumorigenic phenotype. To investigate this further, we performed both *in vitro* and *in vivo* assays. Normal cells require stimulation by mitogenic signals and adhesion to a matrix to switch from a quiescent into a proliferative state. UCMSC cultured in serum deprived medium showed an expected growth arrest, similar to cells that had been γ-irradiated at 40 gray (Figure 6A). Serum deprived HepG2 liver cancer cells, on the contrary, continued to proliferate albeit with a slower rate than cells cultured in a 10% serum supplemented medium. Anchorage-independent growth of UCMSC in soft agar could not be detected in contrast to positive control HepG2 cells that readily formed colonies (Figure 6B). Together, these *in vitro* assays suggested that UCMSC were not tumorigenic.

Finally, we investigated the *in vivo* behavior of UCMSC following subcutaneous transplantation in nude mice. Ten million UCMSC from each donor at P9 or HepG2 cells were injected subcutaneously into (n = 3) 4 weeks-old immunodeficient Balb-c NuNu mice (Figure 6C). Cell viability was assessed on the remaining cell fraction following injection and was of 93.9%±1.8. Mice transplanted with HepG2 cells developed tumors and general signs of illness (anorexia, hunching) two weeks after injection. On the other hand, no tumor was found in mice transplanted with UCMSC (Figure 6C). Moreover, histochemical analysis of the injection site 24 weeks post injection further concluded to the absence of teratoma formation (data not shown).

## Discussion

This study demonstrates that UCMSC isolated from full-term neonates represents a safe source for clinical stem cell therapy as cells reached *in vitro* senescence after long-term culture without any genetic instability or oncogene activation while retaining their differentiation potential.

Here, we analyzed a subpopulation of Wharton’s jelly MSC characterized by a specific immunophenotypic profile. Cells were positive for MSC markers CD29, CD44, CD73, CD90 and CD105 [41] but did not express the CD146 antigen, characteristic of umbilical endothelial cells and pericytes, although CD146 expression was previously detected in a distinct subpopulation of Wharton’s jelly MSC [42]. These discrepancies may be related to the isolation method we have used that did not extract these vascular cells and pericytes from the umbilical cord. UCMSC growth potential was quite homogeneous between the 14 donors we tested and cells were able to undergo an average of 33.7±2.1 population doublings before reaching senescence, consistent with previous reports [42], [43]. Interestingly and in contrast to adult MSC [44], the life span of UCMSC in culture was very similar between samples. Our analyses of telomeres during culture showing, on one hand, a decrease in length and, on the other hand, an increased frequency of telomere dysfunction-induced foci, favor the hypothesis that the life span of UCMSC is dictated by telomere erosion. We do not however completely exclude any involvement of premature entry into senescence, a process that was reported to happen in cells exposed to culture stress [45], as DNA damage foci were also detected outside telomeres at late passages. Recent reports have suggested that a theoretical cell number of 100–200 10^6^ cells/kg - which represents 5–10% of the liver cell mass - should be enough to obtain sufficient metabolic activity to restore the deficient hepatic function [2]. Here we showed that these cell numbers could be obtained between P4 and P6, corresponding to 11–13 CPD. At this stage, transplanted cells still present optimal growth potential to permit liver repopulation.

In line with previous work, UCMSC subjected to a hepatic differentiation protocol acquired a hepatocyte-like phenotype [7]. Analysis of selected hepatocyte markers and metabolic functions confirmed that UCMSC displayed both early and more mature hepatic characteristics. Similarly, we showed that UCMSC were able to undergo both osteogenic and adipogenic differentiation under appropriate conditions. Recent studies have evidenced that the differentiation potential of stem cells progressively declines with aging [31], [32], [46]. Here, we found that, although the multilineage differentiation potential was lost in senescent cells, UCMSC fully retained the ability to differentiate up to advanced passages.

Although still controversial, several reports underlined the risk of human MSC transformation during long-term *ex vivo* culture [13] while others questioned it [44], [47], [48], [49] or findings were later retracted [50], [51]. In this study, we found that UCMSC never bypassed the senescence phase and maintained a stable karyotype. Analyses performed at P9 and at senescence could not evidence any chromosomal abnormality or unbalanced rearrangement in 13/14 donors. UCMSC from donor 2 revealed a polymorphism of chromosome 22 that was not correlated with sub-microscopic allele loss in relevant oncogenic regions. We believe that this polymorphism may already be present in the donor. Alteration of cell cycle regulation and acquired unlimited replicative potential were considered as two key players during the multistep transformation progression of MSC [52]. Cell immortalization requires telomere stabilization and maintenance, which can be achieved by two distinct mechanisms. The first acts through telomerase activation [53]: telomerase is active in some normal stem cells and in about 90% of cancer cells. The second mechanism relies on telomeric DNA recombination for telomere maintenance and is detected mostly in sarcomas and *in vitro* SV40-immortalized fibroblasts [54]. Measurement of telomerase activity, telomerase gene expression and analysis of telomere length profiles in UCMSC at various passages did not reveal any activation of telomere maintenance mechanism. Premalignant alterations of cell cycle regulation genes in UCMSC were further excluded by a series of experiments. First, we showed that the cells expressed stable levels of functional p53 during the whole culture process and maintained both alleles of the *p53* gene on chromosome 17. Second, as expected for normal cells undergoing senescence, p21 and p16 protein levels were upregulated in parallel with increased positive SA-β-gal staining and decreased pRb phosphorylation. Finally, oncogenes such as c-Myc were not induced [55] and UCMSC remained dependent on both anchorage and exogenous growth signals for cell proliferation. *In vivo* experiments corroborated with *in vitro* data as we showed that UCMSC were not able to induce tumor formation in immunocompromised mice models.

### Conclusion

Taken together, our data suggest that UCMSC represent a safe cell source for regenerative medicine. Their origin at the frontier between fetal and adult cells gives them specific biological characteristics of both types but, unlike adult cells, UCMSC have not yet been exposed to cumulative stress inducing DNA damage. Our study showed that UCMSC retain their proliferation and differentiation potential over a long enough period of time to allow sufficient cell expansion to reach numbers needed for stem cell therapy. Furthermore, they undergo normal senescence and fail to adopt tumorigenic potential suggesting that they may represent a safe source for cell therapy.

## Supporting Information

Figure S1 **Post senescent UCMSC cluster characterization.** A. Morphology of a post senescent UCMSC cluster issued from donor 4. B. Post senescent UCMSC were not able to form colonies in Agar Noble. C. FACS analysis on these cells did not reveal any phenotypic modification.
(TIF)

Figure S2 **Hepatogenic** ***in vitro*** **differentiation potential of UCMSC.** A. Optical microscopy pictures showing glycogen storage depicted after Periodic acid-Schiff staining in undifferentiated (−HS) and hepatocyte-like differentiated (+HS) UCMSC at P9 and P18. Human hepatocytes (hHep) were used as a positive control. B. Alizarin Red staining for osteogenic lineage was performed at selected passages (P3–P9–P18–P21). The mineralized area in each image, evidenced by positive stain, was quantified using imageJ. C. Oil Red O staining for adipogenic lineage. The percentage of cells containing lipid droplets was quantified by counting at least 200 cells at indicated passages. Results are mean±SEM of 5 independent experiments. D: differentiated, UD: undifferentiated UCMSC.
(TIF)

Figure S3 **Karyotype and FISH analysis of UCMSC issued from donor 2.** A. The karyotype of donor 2 displayed a polymorphism of chromosome 22. B. Compared G-banding pattern of chromosome 22 showing polymorphism. C-D. Metaphase FISH analysis of chromosome 22 in donor 2. (C) The subtelomere 22q is stained orange and the BCR locus is stained green. The results confirmed the absence of sub-microscopic BCR-ABL translocation. (D) FISH using dual break apart probe of centromeric (XBP1-EWSR1, orange) and telomeric (EWSR1-EPI64, green) flanking regions of EWSR1. The presence of two fusion signals confirmed the absence of EWSR1 rearrangement.
(TIF)
