# Supplementary figures and images for: Human Umbilical Cord Matrix Stem Cells Maintain Multilineage Differentiation Abilities and Do Not Transform during Long-Term Culture
Source: PLoS One. 2013 Aug 9;8(8):e71374. doi: 10.1371/journal.pone.0071374 (PMC3739759; doi:10.1371/journal.pone.0071374)

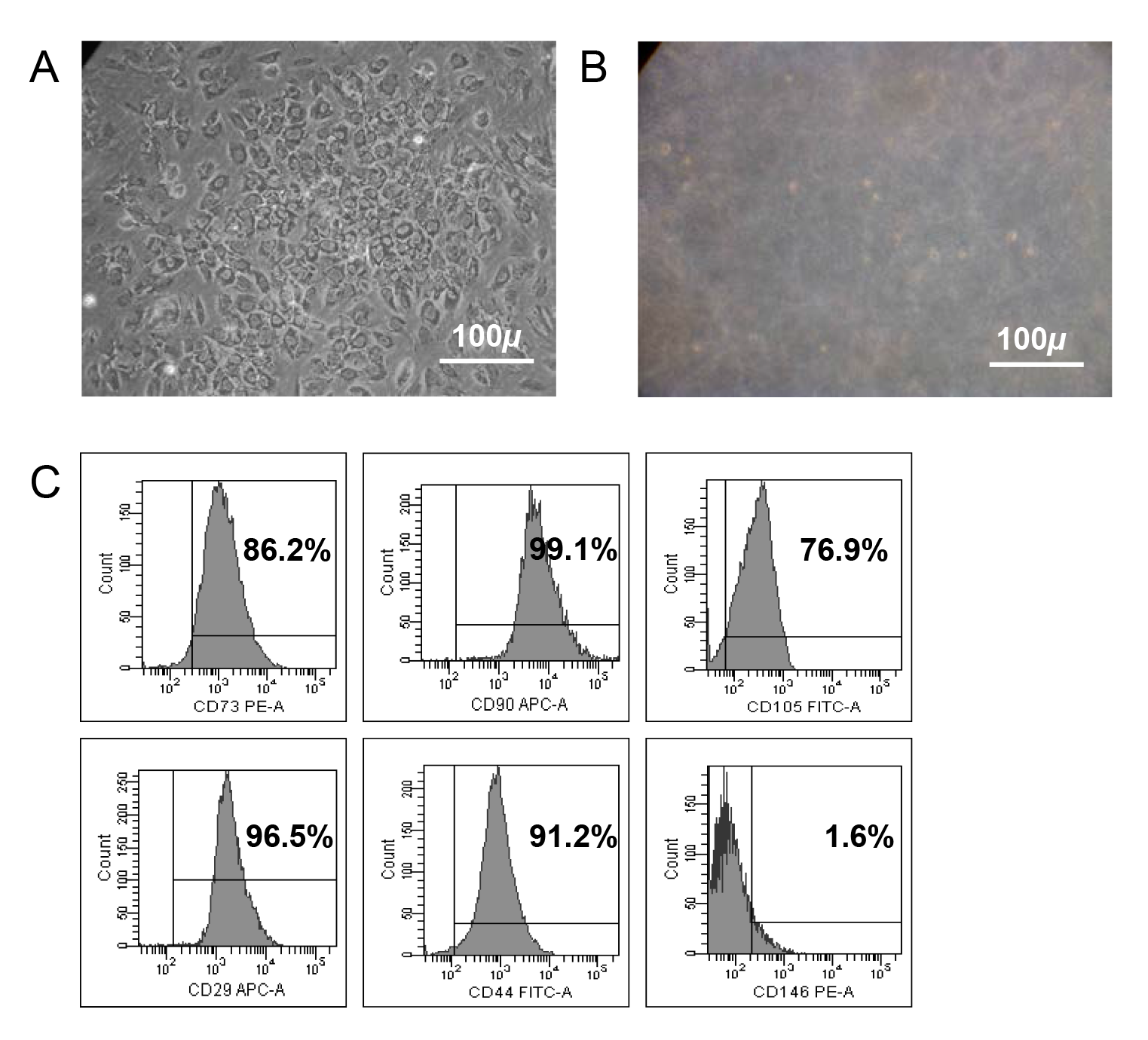

Supplement: Figure S1 — Post senescent UCMSC cluster characterization. A. Morphology of a post senescent UCMSC cluster issued from donor 4. B. Post senescent UCMSC were not able to form colonies in Agar Noble. C. FACS analysis on these cells did not reveal any phenotypic modification. (TIF) [file pone.0071374.s001.tif]

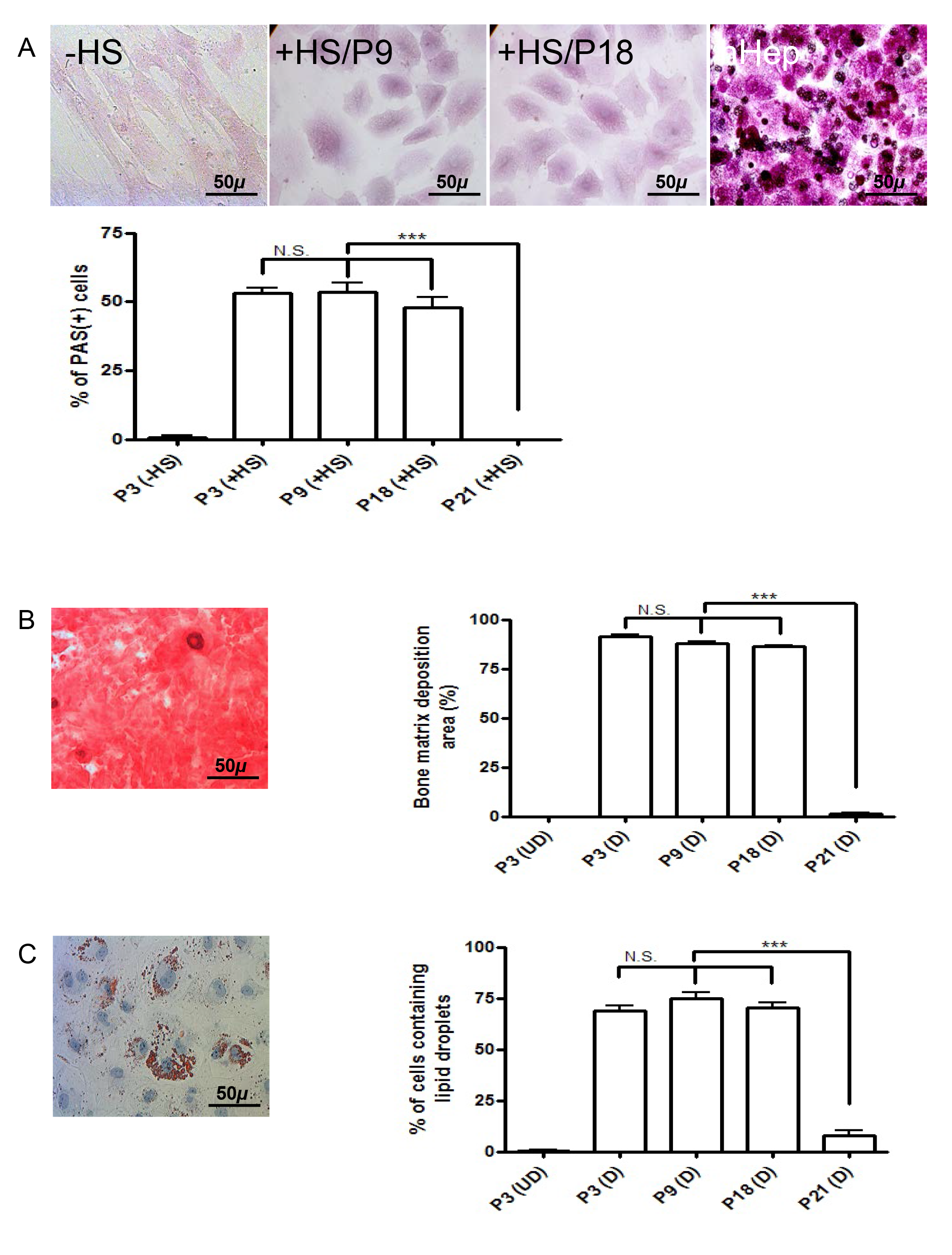

Supplement: Figure S2 — Hepatogenic in vitro differentiation potential of UCMSC. A. Optical microscopy pictures showing glycogen storage depicted after Periodic acid-Schiff staining in undifferentiated (−HS) and hepatocyte-like differentiated (+HS) UCMSC at P9 and P18. Human hepatocytes (hHep) were used as a positive control. B. Alizarin Red staining for osteogenic lineage was performed at selected passages (P3–P9–P18–P21). The mineralized area in each image, evidenced by positive stain, was quantified using imageJ. C. Oil Red O staining for adipogenic lineage. The percentage of cells containing lipid droplets was quantified by counting at least 200 cells at indicated passages. Results are mean±SEM of 5 independent experiments. D: differentiated, UD: undifferentiated UCMSC. (TIF) [file pone.0071374.s002.tif]

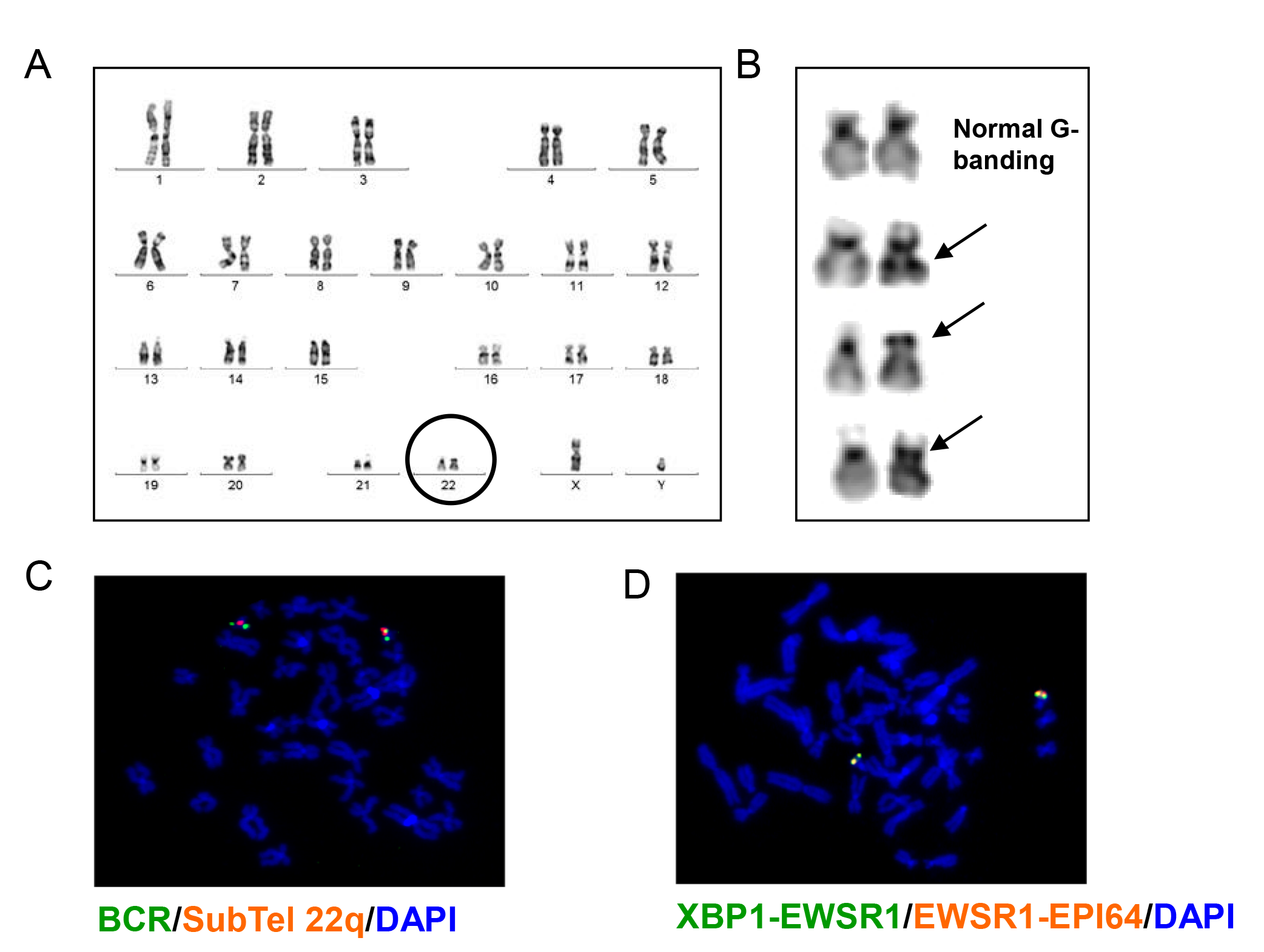

Supplement: Figure S3 — Karyotype and FISH analysis of UCMSC issued from donor 2. A. The karyotype of donor 2 displayed a polymorphism of chromosome 22. B. Compared G-banding pattern of chromosome 22 showing polymorphism. C-D. Metaphase FISH analysis of chromosome 22 in donor 2. (C) The subtelomere 22q is stained orange and the BCR locus is stained green. The results confirmed the absence of sub-microscopic BCR-ABL translocation. (D) FISH using dual break apart probe of centromeric (XBP1-EWSR1, orange) and telomeric (EWSR1-EPI64, green) flanking regions of EWSR1. The presence of two fusion signals confirmed the absence of EWSR1 rearrangement. (TIF) [file pone.0071374.s003.tif]
